# Postoperative quality of life and pain after upper hemisternotomy and conventional median sternotomy for aortic valve replacement: results of a randomized clinical trial

**DOI:** 10.1093/icvts/ivae083

**Published:** 2024-05-15

**Authors:** Idserd D G Klop, Bart P Van Putte, Geoffrey T L Kloppenburg, Robert J M Klautz, Mirjam A G Sprangers, Pythia T Nieuwkerk, Patrick Klein

**Affiliations:** Department of Cardiothoracic Surgery, St Antonius Hospital, Nieuwegein, Netherlands; Department of Cardiothoracic Surgery, St Antonius Hospital, Nieuwegein, Netherlands; Department of Cardiothoracic Surgery, AMC Heart Centre, Amsterdam University Medical Center, Amsterdam, Netherlands; Department of Cardiothoracic Surgery, St Antonius Hospital, Nieuwegein, Netherlands; Department of Cardiothoracic Surgery, AMC Heart Centre, Amsterdam University Medical Center, Amsterdam, Netherlands; Department of Medical Psychology, Amsterdam UMC Location University of Amsterdam, Amsterdam, Netherlands; Department of Mental Health, Amsterdam Public Health, Amsterdam, Netherlands; Department of Medical Psychology, Amsterdam UMC Location University of Amsterdam, Amsterdam, Netherlands; Department of Cardiothoracic Surgery, St Antonius Hospital, Nieuwegein, Netherlands

**Keywords:** Patient-reported outcomes, Cardiac-related quality of life, Upper hemisternotomy, Limited access aortic valve replacement, Randomized trial

## Abstract

**OBJECTIVES:**

Surgical aortic valve replacement through conventional sternotomy yields excellent results. Minimally invasive techniques are deemed equally safe and serve as a viable and less traumatic alternative. However, it is unclear how both surgical techniques affect patient-reported outcomes. The objective of this trial is to compare postoperative cardiac-related quality of life and postoperative pain after upper hemisternotomy and conventional surgical aortic valve replacement.

**METHODS:**

In this single-centre, open-label, investigator-initiated randomized clinical trial, patients were randomized to upper hemisternotomy or conventional full median sternotomy. Patients unable to undergo randomization were monitored prospectively (registry group). Primary outcome was cardiac-specific quality of life, measured with the Kansas City Cardiomyopathy Questionnaire up to 1 year postoperatively.

**RESULTS:**

Patients undergoing upper hemisternotomy had a significantly higher physical limitation domain score across all postoperative time points than patients undergoing conventional surgical aortic valve replacement (estimated mean difference 2.12 points; *P* = 0.014). Patients undergoing upper hemisternotomy were more likely to have a pain score <30 the first 2 days postoperatively than patients undergoing conventional surgical aortic valve replacement (odds ratio 2.63; *P* = 0.007). This was associated with reduced opioid analgesic intake. Postoperative surgical outcome did not differ between both groups.

**CONCLUSIONS:**

Surgical aortic valve replacement through both conventional sternotomy and upper hemisternotomy resulted in clinically similar and important improvements in quality of life, with a small advantage for upper hemisternotomy, while there was no compromise in safety.

## Graphical Abstract

**Figure:**
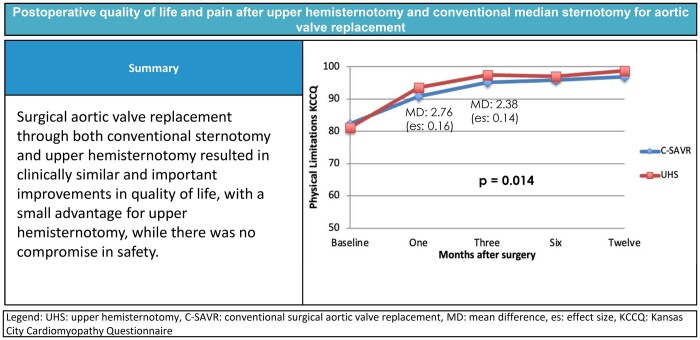

## INTRODUCTION

Aortic valve stenosis remains one of the most common valvular diseases in industrialized countries. Current European Society of Cardiology/European Association for Cardiothoracic Surgery guidelines recommend surgical aortic valve replacement (SAVR) in (a)symptomatic patients with severe aortic valve stenosis at low or intermediate surgical risk [1, 2]. Outcome of SAVR through full median sternotomy (FMS) is excellent in terms of mortality and morbidity, in elective primary cases at low surgical risk (EuroSCORE II < 4) [3, 4]. Minimally invasive or limited access SAVR techniques, such as upper hemisternotomy (UHS) and anterior right thoracotomy, are deemed equally safe and serve as a viable alternative for SAVR through FMS [5].

The aim of minimally invasive SAVR techniques is reduction in surgical trauma, combined with the performance and durability of surgical valve replacement [6], potentially reducing perioperative morbidity. However, minimally invasive SAVR remains subject of debate. While previous studies suggested potential benefits of minimally invasive SAVR in terms of length of hospital stay, blood transfusion and intubation time [7], robust scientific data demonstrating its advantages over conventional SAVR, in terms of quality of life (QoL) and postoperative pain is lacking. One of the reasons for this gap in knowledge is the lack of studies including patient-reported outcomes as their primary end-point [8]. Gaining insight into postoperative QoL is important for supporting decision-making regarding the choice between surgical techniques [9].

The objective of this randomized controlled trial (RCT) is to compare postoperative cardiac-related QoL, generic QoL and postoperative pain after UHS versus conventional SAVR through FMS. The primary hypothesis is that an UHS approach for the treatment of an aortic valve stenosis is associated with a greater improvement in cardiac-related QoL with equal surgical outcomes when compared to conventional SAVR.

## METHODS

### Ethics statement

The Medical Research Ethics Committees United approved the study protocol of the LImited access Aortic valve Replacement (LIAR) trial (NL56311.100.16). The study complies with the Declaration of Helsinki and written informed consent was obtained from all participating patients. The study is registered at ClinicalTrials.gov, number NCT04012060.

### Study design

The LIAR trial is an investigator-initiated, single-centre, open-label RCT conducted at the St Antonius Hospital in the Netherlands.

### Patient selection

Patients were eligible for inclusion if they suffered from severe and/or symptomatic aortic valve stenosis, defined as an aortic valve area of ≤1.0 cm^2^, a mean aortic valve pressure gradient (PG) of ≥40 mmHg and/or a peak velocity of at least 4.0 m/s, and preferred implantation of a biological aortic valve prosthesis, according to standard guidelines [2]. Patients were excluded if they (i) were unable or unwilling to give written informed consent; (ii) were unable to answer the questionnaires due to cognitive or language barriers; (iii) were undergoing concomitant cardiac surgery; (iv) were undergoing a reoperation [as these are only performed through full median (re-)sternotomy]; (v) were unable to undergo a limited access approach; (vi) were undergoing an emergency operation; (vii) had a recent myocardial infarction (<90 days); and/or (viii) had a recent stroke or transient ischaemic attack (<6 months).

### Recruitment and consent

All patients identified by the Heart Team and fulfilling the inclusion criteria were recruited at the outpatient clinic. For those patients willing to participate in the trial, written informed consent was obtained, after which patients were scheduled for surgery. Patients identified as not eligible for participation in the randomized trial were recruited at the outpatient clinic to participate in the prospective registry.

### Randomization and treatment

Randomization was performed by a single research physician in a 1:1 fashion. Randomization took place after the patient was scheduled for operation and, more importantly, after baseline visit and completion of QoL questionnaires. A detailed description of the randomization process has been previously described [10]. Patients were randomized to undergo SAVR through either UHS or conventional sternotomy. UHS was performed using 6–8 cm midline incision, from the angel of Louis to the height of the 3rd or 4th intercostal space. Arterial cannulation was in the distal ascending aorta, while venous cannulation was performed either through the right atrial appendage or common femoral vein. A left ventricular vent was positioned through the right upper pulmonary vein. Alternatively, pulmonary artery venting could be used in case of limited exposure of the right upper pulmonary vein. After clamping of the aorta, cold crystalloid cardioplegia was administered into the aortic root or selectively into the coronary ostia. After oblique aortotomy, removal of the leaflets and decalcification of the annulus was followed by implantation of the aortic valve prosthesis. Re-alignment and closure of the sternum was by means of 3 or 4 single steel wires, with proper re-alignment at the J-point. No sternal plates or pectoral blocks were used during the trial. Conventional SAVR was performed utilizing an 18–20 cm midline incision. Arterial cannulation was at the distal ascending aorta, while venous cannulation was through the right atrial appendage. The remaining procedure is identical to UHS, except for the number of steel wires to close the sternum. Anaesthetic protocol was similar for all patients. Both surgical techniques have been previously described [10].

The randomized allocation of surgical technique was revealed to the surgeons 1 day prior to surgery. Three experienced surgeons (i.e. performing cardiac surgery in approximately >250 patients per year) in both the conventional and limited access technique performed surgery on all randomized patients. The 3 surgeons were experienced with a minimum of at least 50 hemisternotomies for AVR. Two of the 3 surgeons had performed >100 hemisternotomies for AVR, prior to the start of the trial. It is impossible to blind the surgeon, but patients were blinded up to the 4th postoperative day on the nursing ward. This was accomplished by covering the entire length of the sternum with a bandage during that period. The bandage was only changed if deemed necessary by a research nurse. While changing the bandage, patients were asked to close their eyes.

### Data collection and follow-up

Up to 30 days prior to surgery (baseline assessment), and before randomization, the Kansas City Cardiomyopathy Questionnaire (KCCQ) and Medical Outcomes Study Short Form 36 item Health Survey (SF-36) were administered to all patients face-to-face or by telephone, by reading the questions and response options out loud. Baseline patient characteristics were obtained simultaneously. A physician of the research team on the nursing ward obtained postoperative pain scores on day 1 until day 7, or until discharge (in case postoperative length of stay is shorter than 7 days). The same physician monitored the use of analgesic drugs during hospital stay. Postoperatively, the same (oral) analgesics were prescribed to all patients on the nursing ward; morphine and paracetamol. This was part of our postoperative protocol, which was the same for every patient. After 4 days, we stopped prescribing morphine and continued to prescribe paracetamol. Telephone contact was made during follow-up at 1 month (±1 week), 3 months (±30 days), 6 months (±30 days) and 12 months (±60 days). At these moments, the SF-36 and KCCQ were administered and information regarding any adverse events was collected. The same research physician conducted all follow-up assessments.

### Prospective registry

Patients unable or unwilling to participate in the RCT for any reason were monitored in a prospective registry, if written consent was obtained. The registry ran in parallel with the LIAR trial as an integral part. The rationale and design have been previously described [10].

It was the intention to treat all patients participating in the RCT, with an Edwards Intuity Elite rapid deployment stented aortic bioprosthesis, to ensure that only the surgical procedure would differ. If perioperatively, the aortic annulus was too wide or too asymmetrical, increasing the risk of paravalvular leakage (PVL), a sutured valve prosthesis was implanted at the surgeon’s discretion. Patients participating in the prospective registry were treated with either a mechanical or biological sutured valve prosthesis. The choice of valve prosthesis was discussed with the patient prior to surgery.

### Primary outcome measures

The primary outcome was cardiac-related QoL, measured by the physical limitation and symptoms (frequency, severity and recent change over time) domain of the KCCQ across all postoperative time points (i.e. 1, 3, 6, 12 months). The KCCQ consists of 23 items that form 5 domains. All items refer to the past 2 weeks. Scores range from 0 to 100, higher scores indicating a more favourable outcome.

### Secondary quality of life outcome measures

Secondary outcomes included the 3 remaining domains of the KCCQ (self-efficacy, social interference and QoL), generic QoL assessed with the Physical Component Summary (PCS) and the Mental Component Summary (MCS) of the SF-36 and postoperative pain assessed with the Visual Analogue Scale (VAS). Scores of the SF-36 and VAS range from 0 to 100. Higher scores on the SF-36 indicate a more favourable outcome, while a higher VAS score indicates more pain.

### Perioperative outcome

Perioperative outcome included length of incision, prosthetic valve size and technical success rate defined as a limited access approach without conversion and implantation of a rapid deployment bioprosthesis.

### Secondary surgical outcome

Secondary surgical outcomes included aortic cross clamping (ACC) time (min), cardiopulmonary bypass (CPB) time (min) and total operating time (min). Postoperative surgical safety, procedural and performance outcomes were measured by 30-day mortality and 1-year mortality rate, complication rate (according to the VARC3-criteria [11]), hospital length of stay (nights spent in the hospital postoperatively), duration of postoperative mechanical ventilation, length of intensive care unit stay (hours), haemodynamic outcomes (effective orifice area and mean PG in mmHg measured through transthoracic echocardiogram), reoperation rate (number of total reoperations during follow-up) and use of oral analgesic drugs during admission (in milligrams).

### Sample size calculation

The minimal clinically important difference (MCID) for the KCCQ is 5 points. We assumed a standard deviation (SD) of 11 points based on previous literature [12]. To be able to detect at least a 5-point difference between intervention and control group in physical limitations and symptoms assessed with the KCCQ with a two-sided *P*-value of 0.025 and a power of 80%, at least 76 patients per group were required. We decided to include at least 80 patients per group to account for any loss to follow-up and crossovers.

### Statistical analysis

Symptoms and physical limitations domains (KCCQ) were used as multiple primary end-points (i.e. at least 1 statistically significant end-point can be considered a positive result). Differences between intervention and control group in mean scores on these 2 outcomes, across postoperative time points were investigated using mixed linear models. Using a mixed model, based on data from all longitudinal assessments contributing to the treatment comparisons, results in more precise estimates of treatment effects [13]. Models included treatment group (UHS or conventional SAVR), time (1, 3, 6 and 12 months postoperative) and treatment by time interaction as fixed effects, intercept as random effect, to account for repeated measurements within patients, and baseline QoL score and other baseline characteristics as covariates. Insignificant treatment by time interaction was removed from the models. If a statistically significant main effect of treatment group (i.e. mean difference between treatment groups across all postoperative time points) was found, statistical significance of differences between both treatment groups at separate postoperative time-points were interpreted. Effect sizes (Cohen’s d) were calculated by dividing significant mean between group differences by the SD of both groups combined and can be interpreted as small (0.2), medium (0.5) or large (0.8) [14]. Additionally, differences in mean scores on the other KCCQ scales and the PCS and MCS between intervention and control groups over time were investigated using similar linear mixed model regression analyses. We compared the proportion of patients with a VAS score >30 (yes/no) between intervention and control group over time using generalized estimating equations with a binomial distribution, a logit link and unstructured covariance. Generalized estimating equations consider the repeated measurements of pain within patients. The likelihood of having a pain score <30 is expressed as odds ratio. We chose 30 as cut-off value since a score higher than 30 was reason to administer analgesic medication postoperatively.

All continuous data are presented as the mean (SD), whereas categorical data are presented in percentages. All categorical peri- and postoperative outcomes were analysed using the chi-squared test, while all continuous peri- and postoperative outcomes were analysed using the independent samples Student’s *t*-test or Mann–Whitney *U*-test, depending on their distribution. An intention-to-treat analysis as well as a per-protocol analysis were performed.

### Registry group analysis

Postoperative QoL and clinical data were analysed in the same way as the data from the trial patients. Statistical analysis was performed using SPSS version 25.0 (SPSS, Chicago, IL, USA). Two-tailed *P*-values <0.05 were considered statistically significant.

## RESULTS

### Study population

**Figure 1:**
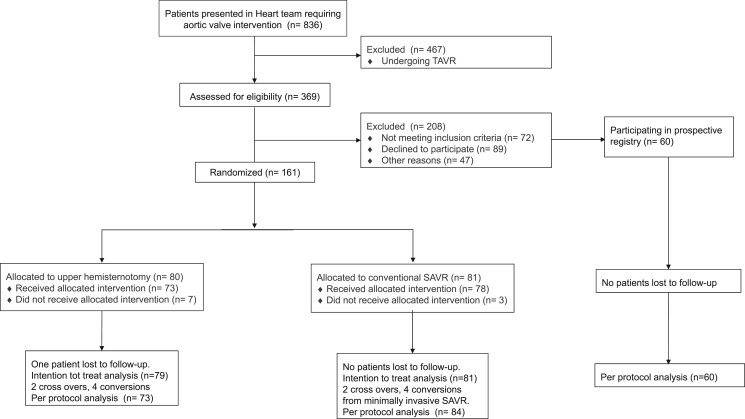
Flow chart of the LIAR trial.

Between July 2016 and April 2019, 369 patients were screened for eligibility. This led to the inclusion of 221 patients. All patients were Caucasian. A total of 161 patients participated in the RCT. An additional 60 patients participated in the prospective registry. One-year follow-up was achieved in 100% and 98.8% of the UHS and conventional SAVR groups, respectively. In the prospective registry, 1-year follow-up was achieved in 93.3% of patients. The Consort Flow chart is shown in Fig. 1.

### Upper hemisternotomy versus conventional surgical aortic valve replacement

Eighty patients were allocated to UHS and 81 to conventional SAVR. Ten patients did not receive the allocated intervention. Four of them were found to have extensive calcifications of the ascending aorta perioperatively, for which surgery was aborted. These patients were scheduled for transcatheter aortic valve replacement (TAVR) during the same admission. Two other patients were crossed over from the UHS group to the conventional SAVR group, due to the sudden unavailability of the trial surgeon (who had to perform emergency surgery) (*n* = 1) or lack of appropriate surgical instruments (*n* = 1). The remaining four patients converted to FMS due to inadequate exposure (*n* = 2), safety (*n* = 1) and inadequate venous drainage (*n* = 1). One patient in the UHS group withdrew from the study postoperatively and is considered lost to follow-up.

### Baseline characteristics

No significant differences were found between randomized groups. Risk of postoperative mortality, estimated by EuroSCORE II, was not significantly different between groups either (Table 1).

**Table 1:** Baseline characteristics.

	Total group, *N* = 161	UHS, *N* = 80	Conventional SAVR, *N* = 81	Prospective registry, *N* = 60	*P*-value UHS versus conventional SAVR
Age	71.6 ± 6.1	72.1 ± 5.9	71.1 ± 6.4	59.8 ± 10.0	0.307
Range, years	57–85	57–85	58–84	41–79	
Female sex	79 (49.1)	42 (52.5)	37 (45.7)	22 (36.7)	0.390
Body mass index	28.1 ± 3.9	28.1 ± 4.2	28.1 ± 3.7	28.2 ± 5.1	0.995
Previous PCI	7 (4.3)	1 (1.3)	6 (7.4)	2 (3.3)	0.056
Diabetes	34 (21.1)	18 (22.5)	16 (19.8)	11 (18.3)	0.672
IDDM	1 (0.6)	0 (0)	1 (1.2)	0 (0.0)	
NIDDM	33 (20.5)	18 (22.5)	15 (18.5)	11 (18.3)	
Previous stroke	2 (1.2)	0 (0)	2 (2.5)	3 (5.0)	0.159
Previous TIA	12 (7.5)	4 (5.0)	8 (9.9)	3 (5.0)	0.241
Active smoker	16 (9.9)	10 (12.5)	6 (7.4)	16 (26.7)	0.283
COPD	18 (11.2)	9 (11.3)	9 (11.1)	4 (6.7)	0.786
GOLD I	2 (1.2)	1 (1.3)	1 (1.2)	2 (3.3)	
GOLD II	14 (8.7)	8 (10.0)	6 (7.4)	2 (3.3)	
GOLD III	2 (1.2)	0 (0)	2 (2.5)	0 (0)	
GOLD IV	0 (0)	0 (0)	0 (0)	0 (0)	
Hypertension	91 (56.5)	44 (55.0)	47 (58.0)	29 (48.3)	0.701
Pulmonary hypertension	17 (10.6)	9 (11.3)	8 (9.9)	4 (6.7)	0.778
Moderate	15 (9.3)	7 (8.8)	8 (9.9)	4 (6.7)	
Severe	2 (1.2)	2 (2.5)	0 (0)	0 (0)	
Kidney function					
Normal	72 (44.7)	33 (41.3)	39 (48.1)	38 (63.3)	0.326
Moderate	80 (49.7)	43 (53.8)	37 (45.7)	22 (36.7)	0.463
Severe	9 (5.6)	4 (5.0)	5 (6.2)	0 (0)	0.841
NYHA					
I	17 (10.6)	12 (15.0)	5 (6.2)	9 (15.0)	0.262
II	101 (62.7)	47 (58.8)	54 (66.7)	34 (56.7)	0.739
III	40 (24.8)	18 (22.5)	22 (27.2)	17 (28.3)	0.525
IV	3 (1.9)	3 (3.8)	0 (0)	0 (0)	0.143
EuroSCORE I (standard)	5.87 ± 1.6	5.93 ± 1.5	5.81 ± 1.6	3.75 ± 1.7	0.654
EuroSCORE I (logistic)	5.27 ± 2.6	5.33 ± 2.5	5.21 ± 2.6	3.01 ± 1.9	0.774
EuroSCORE II	1.38 ± 0.7	1.41 ± 0.8	1.35 ± 0.6	0.96 ± 0.5	0.596

Values presented as mean ± standard deviation or number of patients (percentage).

COPD: chronic obstructive pulmonary disease; IDDM: insulin dependent diabetes mellitus; NIDDM: non-insulin dependent diabetes mellitus; NYHA: new york heart association; PCI: percutaneous coronary intervention; SAVR: surgical aortic valve replacement; TIA: transient ischaemic attack; UHS: upper hemisternotomy.

### Preoperative echocardiographic characteristics

At baseline, almost 86% of patients in the randomized groups had a left ventricular ejection fraction >50%. Mean aortic valve area was 0.8 cm^2^, whereas mean PG was 48 mmHg. In 12.4% of patients, we observed a grade II or higher aortic valve regurgitation in addition to aortic valve stenosis (Supplementary Material, Table S2).

### Perioperative outcomes

A mean incision length of 7.9 cm was needed to perform UHS, which was significantly shorter than the 18 cm needed to perform conventional SAVR (*P* < 0.0001) (Table 2). The Intuity Elite valve prosthesis could be successfully implanted in 138 patients (85.7%). Alternative valve prostheses were implanted for the following reasons: an aortic annulus requiring an—non-available—Intuity Elite size of 29 mm (*n* = 10), a Sievers type 0 bicuspid native aortic valve (*n* = 7), severely calcified ascending aorta for which a TAVR was performed (*n* = 4), severely calcified anterior mitral leaflet deeming it unsafe to implant an Intuity Elite valve (*n* = 1) and an asymmetrical aortic annulus increasing the risk of postoperative PVL (*n* = 1). Implanted valve size was comparable between both groups.

**Table 2:** Perioperative outcomes.

	Total group, *N* = 161	UHS, *N* = 80	Conventional SAVR, *N* = 81	Prospective registry, *N* = 60	*P*-value UHS versus conventional SAVR
Length of incision (cm)	12.9 ± 5.6	7.9 ± 3.0	18.0 ± 1.8	–	<0.0001
Conversion	4 (2.5)	4 (5.0)	–	–	–
Cross over	2 (1.2)	2 (2.5)	0 (0)	0 (0)	–
Implantation Intuity	138 (85.7)	74 (92.5)	64 (79.0)	–	
Transcatheter AVR	4 (2.5)	1 (1.3)	3 (3.7)	0 (0)	
29-mm Intuity	10 (6.2)	5 (6.3)	5 (6.2)	–	
Sievers type 0	7 (4.3)	4 (5.0)	3 (3.7)	–	
Unsafe	1 (0.6)	0 (0)	1 (1.2)	–	
Risk of PVL	1 (0.6)	0 (0)	1 (1.2)	–	
Intercostal space					
Third	48 (29.8)	48 (60.0)	–	–	–
Fourth	31 (19.3)	31 (38.8)	–	–	–
Valve size (mm)	23.8 ± 2.5	23.4 ± 2.4	24.2 ± 2.6	24.2 ± 2.2	0.062
19	9 (5.6)	6 (7.5)	3 (3.7)	0 (0)	
21	32 (19.9)	17 (21.3)	15 (18.5)	11 (18.3)	
23	47 (29.2)	26 (32.5)	21 (25.9)	17 (28.3)	
25	36 (22.4)	19 (23.8)	17 (21.0)	21 (35.0)	
27	26 (16.1)	9 (11.3)	17 (21.0)	7 (11.7)	
29	7 (4.3)	2 (2.5)	5 (6.2)	4 (6.7)	
Valve type					
Crown	3 (1.9)	0 (0)	3 (3.7)	3 (5.0)	
EPIC	4 (2.5)	1 (1.3)	3 (3.7)	9 (15.0)	
Intuity	138 (85.7)	74 (92.5)	64 (79.0)	2 (3.3)	
Inspiris	0 (0)	0 (0)	0 (0)	1 (1.7)	
Magna Ease	11 (6.8)	4 (5.0)	7 (8.6)	6 (10.0)	
Trifecta	1 (0.6)	0 (0)	1 (1.2)	2 (3.3)	
Carbomedics	0 (0)	0 (0)	0 (0)	37 (61.7)	
Bicuspid native valve	46 (28.6)	21 (26.3)	25 (30.9)	28 (46.7)	0.520
Type 0	7 (4.3)	3 (3.8)	4 (4.9)	9 (15.0)	
Type 1a	35 (21.7)	17 (21.3)	18 (22.2)	17 (28.3)	
Type 1 b	4 (2.5)	1 (1.3)	3 (3.7)	2 (3.3)	
Type 1c	0 (0)	0 (0)	0 (0)	0 (0)	
Type 2	0 (0)	0 (0)	0 (0)	0 (0)	
Tricuspid native valve	115 (71.4)	59 (73.8)	56 (69.1)	31 (51.6)	0.520
Monocuspid native valve	0 (0)	0 (0)	0 (0)	1 (1.7)	

Values presented as mean ± standard deviation or number of patients (percentage). *P*-value is the result of minimally invasive SAVR compared with conventional SAVR.

AVR: aortic valve replacement; PVL: paravalvular leakage; SAVR: surgical aortic valve replacement; UHS: upper hemisternotomy.

### Primary outcomes

Intention-to-treat analysis demonstrated that patients undergoing UHS had significantly higher overall postoperative scores on the physical limitation domain of the KCCQ, indicating better QoL, than patients in the conventional SAVR group (96.66 vs 94.54, respectively, estimated mean difference 2.12 points; *P* = 0.014). Analysing the overall effect demonstrated a statistically significant difference at 1 and 3 months postoperatively, but not at 6 and 12 months. This was accompanied by an effect size of 0.16 and 0.14, respectively. No significant difference in the symptoms domain of the KCCQ was found between both groups (Table 3).

**Table 3:** Primary and secondary QoL, intention-to-treat analysis.

UHS versus conventional SAVR in a randomized setting			
Primary outcome (KCCQ)	Mean difference ‘across all’ postoperative time points	*P*-value	Effect size
Physical limitations	2.12 (0.96–3.28)	0.014	
	Mean difference ‘at specific’ post-operative time points		
Month 1	2.76 (0.42–5.10)*		0.16
Month 3	2.38 (0.07–4.68)*		0.14
Month 6	1.19 (–1.10 to 3.49)		
Month 12	1.83 (–0.46 to 4.12)		
	Mean difference ‘across all’ post-operative time points		
Symptoms	2.80 (–0.09 to 5.69)	0.057	
Secondary outcome (KCCQ)			
Quality of life	0.91 (–2.87 to 4.68)	0.64	
Social limitations	2.83 (–0.75 to 6.41)	0.12	
Self-efficacy	–0.31 (–4.30 to 3.67)	0.88	
Secondary outcome (SF36)			
Physical component summary	0.45 (–1.65 to 2.54)	0.67	
Mental component summary	0.78 (–0.83 to 2.40)	0.34	
Postoperative pain			
Postoperative morphine (mg)	–13.61 (–27.91 to 0.69)	0.062	
	Odds ratio		
Pain VAS <30 mm	2.63 (1.30–5.29)	0.007	

Mean difference and odds ratio are presented with their respective 95% confidence intervals. Effect size is given in case 95% confidence interval excludes zero. The asterisk represents the significant differences between both groups at a given time point.

KCCQ: Kansas City Cardiomyopathy Questionnaire; SAVR: surgical aortic valve replacement; SF36: Short Form-36 questionnaire; UHS: upper hemisternotomy; VAS: Visual Analogue Scale.

**Figure 2:**
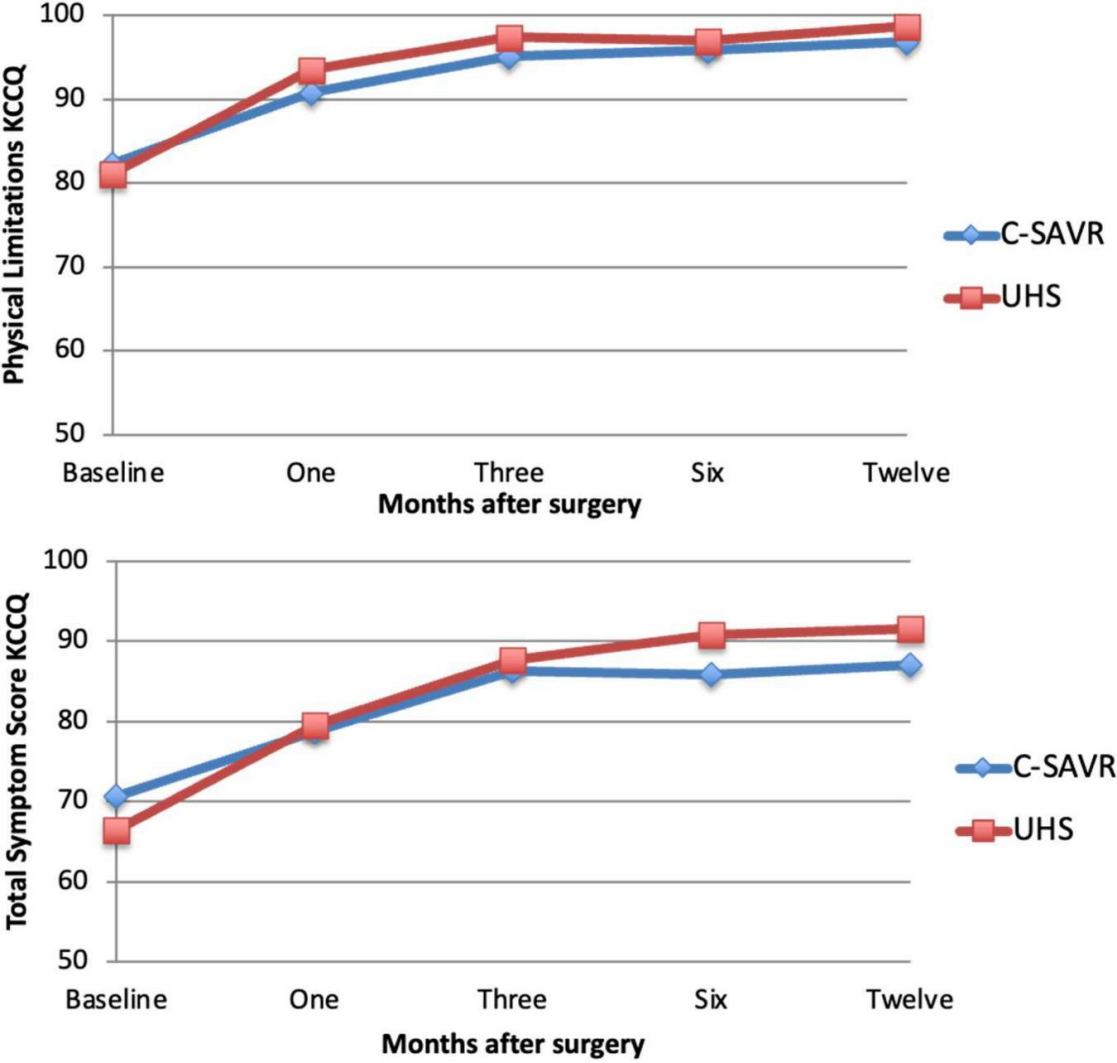
Primary outcomes, physical limitation and total symptom scores from the KCCQ. Higher scores indicate a better QoL. KCCQ: Kansas City Cardiomyopathy Questionnaire; QoL: quality of life; SAVR: surgical aortic valve replacement; UHS: upper hemisternotomy.

Per-protocol analysis confirmed that patients in the UHS group had a significantly higher postoperative physical limitation score (96.89 vs 94.60, respectively, estimated mean difference 2.29 points; *P* = 0.016) 1 and 3 months after surgery (effect size 0.19 and 0.14, respectively). Additionally, patients undergoing UHS had significantly higher postoperative symptoms domain scores (87.73 vs 84.42, respectively, estimated mean difference 3.31 points; *P* = 0.031) at 6 and 12 months postoperatively (Figure 2). This was accompanied by an effect size of 0.27 at both time points (Supplementary Material, Table S3).

### Secondary quality of life outcome

In the intention-to-treat analysis, no differences were seen regarding the other domains of the KCCQ and the SF-36 (Table 2). Per-protocol analysis demonstrated that patients in the UHS group had a significantly higher overall postoperative score on the social limitation domain of the KCCQ (92.13 vs 88.23, respectively, estimated mean difference 3.90 points; *P* = 0.019). The overall effect was significant at 1 month, with an effect size of 0.29. No differences were found in the other domains of the KCCQ or in the PCS and MCS of the SF-36 (Supplementary Material, Table S3).

**Figure 3:**
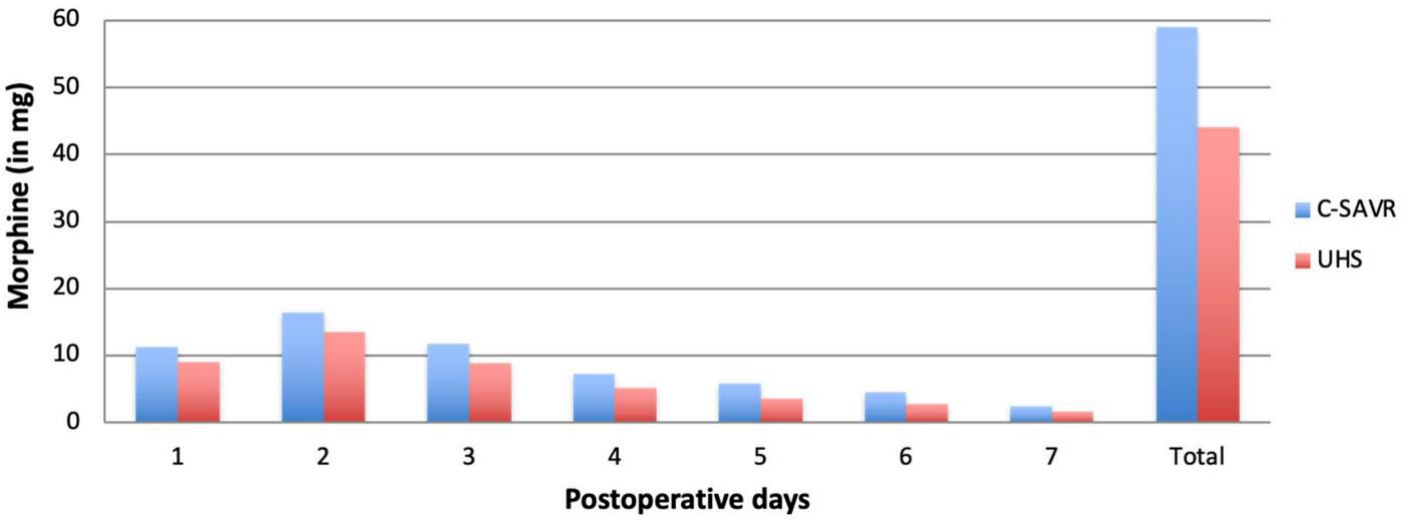
Time frame postoperative administration of morphine. SAVR: surgical aortic valve replacement; UHS: upper hemisternotomy.

Patients undergoing UHS were more likely to have a postoperative pain score <30 on the VAS during the first 2 days at the nursing ward than patients in the C-SAVR group (OR 2.63; *P* = 0.007) in the intention-to-treat analysis. On day 1 postoperatively, the mean difference of both UHS and conventional SAVR was 11.1 points [28.6 (SD 2.2) vs 39.7 (SD 2.2), respectively, *P* = 0.019] and on day 2 postoperatively, the mean difference between both groups was 7.7 points [24.7 (SD 2.3) vs 32.4 (SD 2.2), respectively, *P* = 0.019]. However, the intake of postoperative analgesic medication did not significantly differ between both groups. Patients undergoing UHS had a total opioid use of 44 mg during admission, versus 58 mg for patients undergoing conventional SAVR (mean difference –13.61 mg; *P* = 0.062) (Figure 3). Per-protocol analysis demonstrated that patients in the UHS group were significantly more likely to have postoperative pain scores <30 the first 3 days after surgery (odds ratio 2.79; *P* = 0.006). This was accompanied by reduction in postoperative opioid intake (43 vs 57 mg, respectively, mean difference –13.94 mg; *P* = 0.001). No significant differences were seen between both groups regarding paracetamol administration in either the intention-to-treat or per-protocol analysis.

### Secondary surgical outcome

Patients undergoing UHS had comparable CPB and ACC times compared to conventional SAVR. However, total surgical time was prolonged in patients undergoing UHS [160.7 (SD 59.0) vs 137.5 (SD 45.7 min), *P* = 0.006]. Furthermore, UHS was associated with less postoperative blood loss [369 (SD 163) vs 515 (SD 407 ml), *P* = 0.004]; however, this was not accompanied by a reduction in postoperative administration of blood products. An overview of all surgical outcomes is given in Table 4.

**Table 4:** Secondary surgical outcome, intention-to-treat analysis.

	Total group, *N* = 161	UHS, *N* = 80	Conventional SAVR, *N* = 81	*P*-value
CPB time (min)	72.1 ± 25.4	73.7 ± 21.7	70.5 ± 28.7	0.437
ACC time (min)	48.6 ± 19.8	50.0 ± 17.6	47.2 ± 21.7	0.381
Total time of surgery (min)	148.9 ± 53.9	160.7 ± 59.2	137.5 ± 45.7	0.006
Total time OR (min)	210.2 ± 55.6	223.0 ± 60.9	197.8 ± 47.1	0.004
Blood loss (ml)	443 ± 319	369 ± 163	515 ± 407	0.004
Postoperative transfusion	7 (4.3)	4 (5.0)	3(3.7)	0.787
Ventilation on ICU (hours)	5.6 ± 3.8	4.8 ± 3.7	6.3 ± 3.8	0.067
Length of stay (days)	8 [6–10]	8 [6–11]	7 [6–10]	0.592
30-day mortality	3 (1.9)	0 (0)	3 (3.7)	0.082
Stroke	3 (1.9)	2 (2.5)	1 (1.2)	0.553
Myocardial infarction	0 (0)	0 (0)	0 (0)	–
Acute kidney injury	9 (5.6)	6 (7.5)	3 (3.7)	0.328
LCOS	3 (1.9)	1 (1.3)	2 (2.5)	1.000
Endocarditis	1 (0.6)	0 (0)	1 (1.2)	1.000
Permanent pacemaker	14 (8.7)	8 (10.0)	6 (7.4)	0.402
Third degree AVB	20 (12.4)	12 (15.0)	8 (9.9)	0.324
Sepsis	4 (2.5)	1 (1.3)	3 (3.7)	0.620
Multi-organ failure	2 (1.2)	0 (0)	2 (2.5)	0.497
Major vascular complication	1 (0.6)	1 (1.3)	0 (0)	0.497
Sternal dehiscence	2 (1.2)	1 (1.3)	1 (1.2)	1.000
Mediastinitis	3 (1.9)	2 (2.5)	1 (1.2)	0.620
Severe paravalvular leak	2 (1.2)	1 (1.3)	1 (1.2)	1.000
Readmission ICU	2 (1.2)	1 (1.3)	1 (1.2)	1.000
Tamponade	7 (4.3)	5 (6.3)	2 (2.5)	0.277
Reoperation for bleeding	3 (1.9)	0 (0)	3 (3.7)	0.245
New onset AF	52 (32.3)	28 (35.0)	24 (29.6)	0.466
Left bundle branch block	50 (31.1)	24 (30.0)	26 (32.1)	0.774
Right bundle branch block	4 (2.5)	3 (3.8)	1 (1.2)	0.367

Values presented as mean ± standard deviation, median with interquartile range or number of patients (percentage).

ACC: aortic cross clamping; AF: atrial fibrillation; AVB: atrioventricular block; CPB: cardiopulmonary bypass; ICU: intensive care unit; LCOS: low cardiac output syndrome; OR: operating room; SAVR: surgical aortic valve replacement; UHS: upper hemisternotomy.

### Mortality

Overall, 30-day mortality was 0% for the UHS group and 3.7% for the conventional SAVR group (*P* = 0.082). Of the 3 deaths that occurred, 2 were non-cardiovascular: 1 patient died 24 days after surgery due to a caecum perforation (unknown cause) causing sepsis and multi-organ failure and 1 patient died 12 days after surgery due to mediastinitis causing sepsis and multi-organ failure. The 3rd patient died 9 days after surgery due to ventricular fibrillation. Overall, 1-year survival was 97.5%. During follow-up, 1 patient in the UHS group, suffering from pancreatic cancer, died approximately 9 months after surgery. No patients in the conventional SAVR group died during follow-up.

### Major adverse events

Two patients undergoing UHS and 1 patient undergoing conventional SAVR suffered from a periprocedural ischaemic stroke (1.9%), of which 2 recovered fully. Reintervention rate for bleeding was 0% in the UHS group and 3.7% in the conventional SAVR group (*P* = 0.245). One patient (1.2%) in the conventional SAVR group suffered from an endocarditis (*Staphylococcus lugdunensis*) 20 days after surgery, which was treated successfully with antibiotics. One patient (1.3%) in the UHS group had a major vascular complication. Perforation of the iliac vein with the guide wire of the venous cannula caused an abdominal bleeding. Two patients (1.2%), 1 in each group, suffered from severe PVL, requiring a reoperation within 30 days after surgery.

### Conduction disorders

Postoperative conduction disorders were comparable between both groups. A third-degree atrioventricular block was seen in 20 patients (12.4%). Fourteen (8.7%) of them needed implantation of a permanent pacemaker. New onset atrial fibrillation was seen in 32.3% of the patients, a new left bundle branch block in 31.1% of the patients. We separately analysed the data for small valve sizes (19 and 21 mm) and did not find an increased rate of conduction disorders, pacemaker implantation or PVL. Postoperative echocardiographic haemodynamic outcomes were satisfactory.

### Prospective registry group versus upper hemisternotomy and conventional surgical aortic valve replacement

#### Baseline characteristics

Patients participating in the prospective registry had a mean age of 59.8 (SD 10.0) years, which was significantly lower than patients in the randomized groups [71.6 (SD 6.1), *P* < 0.001]. Additionally, the EuroSCORE II was 0.96 (SD 0.5) vs 1.38 (SD 0.7) in the randomized groups (*P* < 0.001). See Supplementary Material, Table S1 for a full overview.

#### Preoperative echocardiographic characteristics

At baseline, 88.3% of the patients had a left ventricular ejection fraction >50%. Mean aortic valve area was 0.85 cm^2^, whereas mean PG was 46 mmHg (Supplementary Material, Table S2).

#### Perioperative outcome

All patients underwent SAVR through conventional FMS. Mean valve size of the prosthetic valves was 24.2 mm (SD 2.2). Patients were treated with either a biological (38.3%) or mechanical aortic valve prosthesis (61.7%) (Table 2).

#### Primary outcome

Patients in the prospective registry had a significantly lower overall postoperative physical limitation score than patients in the UHS group (estimated mean difference 3.25 points; *P* = 0.016). Analysing the overall effect resulted in a significant difference 1 month after surgery, with an effect size of 0.33. No difference was found between the prospective registry and the UHS group in terms of the symptoms domain. When comparing the prospective registry group to the randomized conventional SAVR group, no differences were seen in the physical limitation and symptoms domain of the KCCQ (Supplementary Material, Table S3).

### Secondary quality of life outcome

Patients in the prospective registry had significantly lower postoperative social limitation scores (estimated mean difference of 6.04 points; *P* = 0.019) than patients in the UHS group. This difference was statistically significant at 1 month postoperatively, with an effect size of 0.49. No differences were seen in the other domains of the KCCQ or in the PCS and MCS of the SF-36. Mean QoL scores for all treatment groups across all time points are given in Supplementary Material, Table S4.

Patients in the prospective registry were significantly less likely to have postoperative pain scores <30 compared to the UHS group (odds ratio 3.28; *P* = 0.006). Mean postoperative pain scores are presented in Supplementary Material, Table S5. This was accompanied by an increase in postoperative administration of opioids in the prospective registry (62 vs 44 mg, respectively, mean difference –18.1 mg; *P* = 0.031).

The prospective registry and conventional SAVR group had comparable scores on all secondary outcomes.

#### Secondary surgical outcome

A full overview of postoperative results of the prospective registry group is given in Supplementary Material, Table S6.

## DISCUSSION

In this single-centre, open-label, RCT, we found better postoperative cardiac-related QoL and lower postoperative pain in favour of patients undergoing SAVR through UHS. The significant differences between UHS and conventional AVR were of a very small magnitude and about half of what we would consider a meaningful difference (e.q. MCID 5 points).

To punctuate the results and give more insight into the generalizability of our data, the prospective registry was added. Comparing patients randomized to UHS with patients in the prospective registry not blinded for treatment undergoing SAVR through conventional FMS, we found QoL benefits for the UHS group. This finding is somewhat surprising, since patients participating in the prospective registry were significantly younger and had lower mean EuroSCORE II. We expected them to have at least comparable QoL at an earlier moment postoperatively. We hypothesized that younger patients expect to recover faster and have good postoperative QoL relatively soon. Conversely, elderly patients may be more aware of their limitations and may have lower expectations about QoL and recovery after surgery. The results might be reinforced by the fact that patients in the prospective registry were still having a job and were more active socially and physically.

Instead of focusing primarily on surgical outcome, including cardiac-related PROs as primary or secondary outcomes in clinical trials is important to gain insight into patients’ functional status and overall QoL [15]. This aids clinicians and patients in making evidence-based treatment decisions. Few studies have investigated generic QoL, but none have investigated cardiac-related or disease-specific QoL. Borger *et al.* [16] demonstrated good generic QoL after minimally invasive SAVR with a rapid deployment stented bioprosthesis, utilizing the EuroQoL five dimensions questionnaire (EQ-5D). No differences were found between minimally invasive SAVR and conventional SAVR. More recently, Rodríguez-Caulo *et al.* [17] published a well-designed RCT, demonstrating significant benefit in favour of minimally invasive SAVR, when compared to SAVR through FMS, regarding generic QoL 1 month after surgery. They also assessed QoL using the EQ-5D and measured patient satisfaction, but not cardiac-related QoL.

At the time our study was designed, no QoL questionnaire specifically focusing on aortic valve stenosis was available. We chose to use the KCCQ as our main cardiac-related QoL questionnaire, as we deemed the KCCQ questions most relevant for our purpose in the absence of an aortic valve stenosis-specific questionnaire. Recently, an aortic valve stenosis-specific questionnaire, the Toronto Aortic Stenosis Quality of life questionnaire (TASQ), has been designed [18]. The TASQ focuses primarily on current limitations patients experience due to their aortic valve stenosis. Instead, the KCCQ focuses on the symptoms of cardiac failure during the last 2 weeks. Additionally, the KCCQ has multiple questions specifically designed for heart failure, which are not applicable to all patients with severe aortic valve stenosis. Consequently, the KCCQ may have been less sensitive to detect differences in QoL among patients participating in the present study.

Nevertheless, both groups demonstrated improvement in postoperative cardiac-related QoL, measured with the KCCQ. The intention-to-treat analysis showed small, but significant improvement in the physical limitations and symptoms domain of the KCCQ, in favour of the UHS group. However, differences were smaller than the previously established MCID of the KCCQ (5 points) [19]. Additionally, effect sizes were generally small, which limits clinical relevance. Per-protocol analysis also showed slightly better QoL in the UHS group than in the conventional SAVR group, but, again, with differences smaller than the MCID and mostly small effect sizes. The slightly better QoL can potentially be explained by the conversions and crossovers in our study.

Reduction of postoperative pain has always been one of the focal points of minimally invasive SAVR. Multiple cohort studies have been performed [20, 21] suggesting a significant reduction in postoperative pain, while a more recent meta-analysis was not able to show a benefit in favour of minimally invasive SAVR [22]. Our data support reduction of postoperative pain after UHS. In this open-label trial, patients were blinded for their treatment the first 4 days postoperatively. This means pain scores taken from patients during the first 4 days were blinded. Not only pain was reduced, but total opioid use after surgery was significantly lower as well in the per-protocol analysis. Most likely, the amount of sternal retraction seems a plausible explanation for both short-term benefits in reduced pain perception but may also be related to longer lasting benefit. Furthermore, postoperative administration of opioids is standard regimen, only if patients experience moderate pain (VAS >30). This means opioid intake is a good representation of postoperative experience of pain.

Despite the reduction in pain and reduced intake of opioids, no significant differences were seen between both groups in terms of postoperative ventilation, intensive care unit stay and total length of hospital stay. We found a median length of hospital stay of 8 days. This is longer than we expected and has multiple causes. One of the reasons is a strict postoperative protocol for mobilization and diagnostics in our centre. Additionally, we are a tertiary centre getting referrals from hospitals throughout the entire county. These hospitals have their own different protocols for discharging patients home.

We did not find any significant differences between both randomized groups regarding physical and mental functioning up to 1 year postoperatively, measured with the PCS and MCS of the SF-36, respectively. These QoL scores were low at baseline but increased significantly after 3 months postoperatively. This is in line with previously reported studies [23].

Limited access SAVR is competing with TAVR, as it is a truly minimally invasive treatment modality and therefor a less traumatic treatment option for patients with aortic valve stenosis. Current debate concerns the treatment of low-risk patients. Three recent randomized trials studied TAVR in low-risk patients [24–26]. Mortality and stroke outcomes strongly favoured TAVR after 1-year of follow-up; however, this benefit was diminished at 2 years. Concerns remain of the association of TAVR with significantly higher permanent pacemaker implantation rates and moderate or severe PVL [26]. Moreover, it is important to note that none of these trials included patients with bicuspid aortic valves, which constituted 28.6% of the patients in our trial. Data on long-term results of TAVR and valve durability, especially in low-risk patients, is still awaited. For patients with small valve sizes, structural valve deterioration occurring during follow-up is a potential, significant problem. Valve-in-valve TAVR procedures are not common practice in patients with smaller valve prostheses, increasing the risk of a surgical reintervention (either through UHS or FMS) at a later age. With 25.5% of the patients in our trial with prosthetic valve sizes of 19 or 21 mm, this is a relevant issue. Because of the minimally invasive nature of TAVR and avoidance of a (partial) sternotomy or thoracotomy, patients typically experience a larger improvement in health status as measured by the KCCQ during the first 30 days postoperatively. However, these differences in KCCQ scores disappeared 1 year after the intervention [25].

Critics of minimally invasive SAVR techniques point to increased complexity and prolonged operative times, without reproducible improvements in morbidity and mortality [27, 28]. Our study indicates UHS can be technically more challenging, demonstrated by prolonged operative time and conversion rate. We suggest that, since ACC and CPB times were comparable between both groups, additional time is needed for gaining adequate exposure, peripheral (de)cannulation, haemostasis and closure. However, this did not increase the risk of postoperative mortality and morbidity. The debate is fuelled by data on minimally invasive SAVR derived from non-randomized studies. Adequately powered RCTs are scarce. One RCT that has been performed is the Mini-Stern trial by Nair *et al.* [29]. The primary results demonstrated that SAVR through upper mini-sternotomy did not result in reduced length of hospital stay, faster recovery or improved survival relative to full sternotomy, while prolonging operative times. Our results confirm that length of stay and survival rate are comparable between both UHS and conventional SAVR. This was accompanied by comparable ACC and CPB times between both groups. A possible explanation can be that the use of rapid deployment valve prostheses contributes to the reduction in ACC and CPB times. Nair *et al.* [29] utilized only sutured valve prosthesis for both the mini-sternotomy group as well as the full sternotomy group. It has been previously described that minimally invasive procedures, combined with sutured valve prostheses, are associated with significantly prolonged operative times [7]. The use of the Intuity Elite rapid deployment prosthesis does come with a price. There seems to be an increased rate of permanent pacemaker implantation, when compared to conventional sutured prostheses [30]. Finally, postoperative blood loss was significantly reduced in the UHS group. This was not associated with a reduction in postoperative transfusion requirement, which corresponds well with a more recently published RCT focusing primarily on postoperative red cell transfusion [6].

### Strengths and limitations

Major strength of the present study is the exceptionally high response rate in both randomized groups as in the prospective registry group. The registry group makes it possible to better generalize and understand our results. Furthermore, the prospective registry provides an overview of postoperative QoL in non-randomized patients. We performed an intention-to-treat analysis, as well as a per-protocol analysis, to present a complete reflection of our results. During the first 4 days on the nursing ward, patients were blinded for their treatment, limiting interference of bias in postoperative pain scores. The outcome measures are validated QoL questionnaires, taken directly from patients, either face-to-face or through telephone interview.

A limitation of our study is multiple testing for many different QoL domains. It is possible that the secondary results favour UHS by chance. Furthermore, the original sample size was calculated based on 1 primary end-point (*P* = 0.05) [10]. Because using multiple primary end-points increases the chance of a type I error, but not a type II error, we have adjusted our *P*-value for multiple testing to 0.025 instead, to indicate statistical significance. This is a minor deviation from our original rationale and design. Notably, the primary end-point physical limitations would still be statistically significant. Thus, this would not have led to different conclusions. Ideally, we would have included 93 patients in the control and intervention group. Despite this power constraint, we were able to detect multiple significant differences. Another limitation is that we measured improvement in heart failure symptoms as outcome, while the only difference between both randomized groups is related to surgical trauma. Furthermore, the used MCID of the KCCQ is not validated for these specific procedures (SAVR through conventional FMS and UHS). This makes it harder to interpret the results. A significant number of patients did not undergo the allocated treatment, which was mitigated by the per-protocol analysis. The QoL questionnaires were administered by telephone by the same research physician, a time-consuming procedure. Moreover, the research physician may have introduced unconscious bias. However, this also means that there is no variability in the way the questionnaires were administered. Additionally, patients were only blinded during the first 4 days after surgery, potentially leading to socially desirable answers and introducing additional bias during administration of the questionnaires at follow-up. This procedure will not affect the internal validity of the results, however, since it is used in both randomized groups and in the registry patients. Due to the single-centre design, the external validity of the LIAR trial is potentially limited. Experienced surgeons, past their learning curve, performed minimally invasive SAVR; therefore, the results might differ in centres with less-experienced surgeons.

## CONCLUSION

SAVR through both conventional sternotomy and UHS resulted in clinically similar and important improvements in QoL, with a small advantage for UHS, while there was no compromise in safety. UHS is a viable alternative for SAVR by conventional median sternotomy and can be performed safely in dedicated hands.

## Supplementary Material

ivae083_Supplementary_Data

## Data Availability

The data underlying this article will be shared on reasonable request to the corresponding author.

## Notes

Presented at the 35th EACTS annual meeting on October 16th 2021, Barcelona, Spain.

## ABBREVIATIONS

ACC: Aortic cross clamping
CPB: Cardiopulmonary bypass
EQ-5D: EuroQoL five dimensions questionnaire
FMS: Full median sternotomy
KCCQ: Kansas City Cardiomyopathy Questionnaire
LIAR: LImited access Aortic valve Replacement
MCS: Mental component summary
MCID: Minimal clinically important difference
PCS: Physical component summary
PG: Pressure gradient
PVL: Paravalvular leakage
QoL: Quality of life
RCT: Randomized controlled trial
SAVR: Surgical aortic valve replacement
SD: Standard deviation
SF-36: Short Form-36
TASQ: Toronto Aortic Stenosis Quality of life questionnaire
TAVR: Transcatheter aortic valve replacement
UHS: Upper hemisternotomy
VAS: Visual analogue scale

## References

[1] Thaden JJ , Nkomo VT, Enriquez-Sarano M. The global burden of aortic stenosis. Prog Cardiovasc Dis 2014;56:565–71. 24838132 10.1016/j.pcad.2014.02.006

[2] Baumgartner H , Falk V, Bax JJ, De Bonis M, Hamm C, Holm PJ et al; ESC Scientific Document Group. 2017 ESC/EACTS Guidelines for the management of valvular heart disease. Eur Heart J 2017;38:2739–91. 28886619 10.1093/eurheartj/ehx391

[3] Kang DH , Park SJ, Lee SA, Lee S, Kim DH, Kim HK et al Early surgery or conservative care for asymptomatic aortic stenosis. N Engl J Med 2020;382:111–9. 31733181 10.1056/NEJMoa1912846

[4] Bowdish ME , D’Agostino RS, Thourani VH, Desai N, Shahian DM, Fernandez FG et al The Society of Thoracic Surgeons Adult Cardiac Surgery Database: 2020 update on outcomes and research. Ann Thorac Surg 2020;109:1646–55. 32247780 10.1016/j.athoracsur.2020.03.003

[5] Ghanta RK , Lapar DJ, Kern JA, Kron IL, Speir AM, Fonner E Jr, et al Minimally invasive aortic valve replacement provides equivalent outcomes at reduced cost compared with conventional aortic valve replacement: a real-world multi-institutional analysis. J Thorac Cardiovasc Surg 2015;149:1060–5. 25680751 10.1016/j.jtcvs.2015.01.014 PMC4409485

[6] Hancock HC , Maier RH, Kasim AS, Mason JM, Murphy GJ, Goodwin AT et al Mini-sternotomy versus conventional sternotomy for aortic valve replacement. J Am Coll Cardiol 2019;73:2491–2. 31097171 10.1016/j.jacc.2019.03.462

[7] Phan K , Xie A, Di Eusanio M, Yan TD. A meta-analysis of minimally invasive versus conventional sternotomy for aortic valve replacement. Ann Thorac Surg 2014;98:1499–511. 25064516 10.1016/j.athoracsur.2014.05.060

[8] Noyez L , de Jager MJ, Markou AL. Quality of life after cardiac surgery: underresearched research. Interact CardioVasc Thorac Surg 2011;13:511–4. 21807814 10.1510/icvts.2011.276311

[9] De Heer F , Gökalp AL, Kluin J, Takkenberg JJM. Measuring what matters to the patient: health related quality of life after aortic valve and thoracic aortic surgery. Gen Thorac Cardiovasc Surg 2019;67:37–43. 28905303 10.1007/s11748-017-0830-9 PMC6323078

[10] Klop IDG , van Putte BP, Kloppenburg GTL, Sprangers MAG, Nieuwkerk PT, Klein P. Comparing quality of life and postoperative pain after limited access and conventional aortic valve replacement: design and rationale of the LImited access aortic valve replacement (LIAR) trial. Contemp Clin Trials Commun, 2021;21:100700. 33506139 10.1016/j.conctc.2021.100700 PMC7815656

[11] Généreux P , Piazza N, Alu MC, Nazif T, Hahn RT, Pibarot P et al; VARC-3 WRITING COMMITTEE. Valve Academic Research Consortium 3: updated endpoint definitions for aortic valve clinical research. Eur Heart J 2021;42:1825–57. 33871579 10.1093/eurheartj/ehaa799

[12] Thourani VH , Suri RM, Gunter RL, Sheng S, O’Brien SM, Ailawadi G et al Contemporary real-world outcomes of surgical aortic valve replacement in 141,905 low-risk, intermediate-risk, and high-risk patients. Ann Thorac Surg 2015;99:55–61. 25442986 10.1016/j.athoracsur.2014.06.050

[13] Detry MA , Ma Y. Analyzing repeated measurements using mixed models. JAMA 2016;315:407–8. 26813213 10.1001/jama.2015.19394

[14] Cohen J. Statistical Power Analysis for the Behavioral Sciences. Academic Press, New York, New York, United States of America, 2013.

[15] Black N. Patient reported outcome measures could help transform healthcare. BMJ 2013;346:f167. 23358487 10.1136/bmj.f167

[16] Borger MA , Moustafine V, Conradi L, Knosalla C, Richter M, Merk DR et al A randomized multicenter trial of minimally invasive rapid deployment versus conventional full sternotomy aortic valve replacement. Ann Thorac Surg 2015;99:17–25. 25441065 10.1016/j.athoracsur.2014.09.022

[17] Rodríguez-Caulo EA , Guijarro-Contreras A, Guzón A, Otero-Forero J, Mataró MJ, Sánchez-Espín G et al Quality of life after ministernotomy versus full sternotomy aortic valve replacement. Semin Thorac Cardiovasc Surg 2021;33:328–34. 32853740 10.1053/j.semtcvs.2020.07.013

[18] Styra R , Dimas M, Svitak K, Kapoor M, Osten M, Ouzounian M et al Toronto aortic stenosis quality of life questionnaire (TASQ): validation in TAVI patients. BMC Cardiovasc Disord 2020;20:209. 32370791 10.1186/s12872-020-01477-2 PMC7201733

[19] Spertus J , Peterson E, Conard MW, Heidenreich PA, Krumholz HM, Jones P et al; Cardiovascular Outcomes Research Consortium. Monitoring clinical changes in patients with heart failure: a comparison of methods. Am Heart J 2005;150:707–15. 16209970 10.1016/j.ahj.2004.12.010

[20] Filip G , Bryndza MA, Konstanty-Kalandyk J, Piatek J, Wegrzyn P, Ceranowicz P et al Ministernotomy or sternotomy in isolated aortic valve replacement? Early results. Kardiochir Torakochirurgia Pol 2018;15:213–8. 30647743 10.5114/kitp.2018.80916 PMC6329886

[21] Candaele S , Herijgers P, Demeyere R, Flameng W, Evers G. Chest pain after partial upper versus complete sternotomy for aortic valve surgery. Acta Cardiol 2003;58:17–21. 12625490 10.2143/AC.58.1.2005254

[22] Kirmani BH , Jones SG, Muir A, Malaisrie SC, Chung DA, Williams RJ et al Limited versus full sternotomy for aortic valve replacement. Cochrane Database Syst Rev 2017;12:CD011793. 10.1002/14651858.CD011793.pub2 PMC6478148 28394022

[23] Reynolds MR , Magnuson EA, Wang K, Thourani VH, Williams M, Zajarias A et al; PARTNER Trial Investigators. Health-related quality of life after transcatheter or surgical aortic valve replacement in high-risk patients with severe aortic stenosis: results from the PARTNER (Placement of AoRTic TraNscathetER Valve) Trial (Cohort A). J Am Coll Cardiol 2012;60:548–58. 22818074 10.1016/j.jacc.2012.03.075

[24] Thyrefid HG , Steinbrüchel DA, Ihlemann N, Nissen H, Kjeldsen BJ, Petursson P et al Transcatheter versus surgical aortic valve replacement in patients with severe aortic valve stenosis: 1-year results from the all-comers NOTION randomized clinical trial. J Am Coll Cardiol 2015;65:2184–94. 25787196 10.1016/j.jacc.2015.03.014

[25] Mack MJ , Leon MB, Thourani VH, Makkar R, Kodali SK, Russo M et al; PARTNER 3 Investigators. Transcatheter aortic-valve replacement with a balloon-expandable valve in low-risk patients. N Engl J Med 2019;380:1695–705. 30883058 10.1056/NEJMoa1814052

[26] Popma JJ , Deeb GM, Yakubov SJ, Mumtaz M, Gada H, O’Hair D et al; Evolut Low Risk Trial Investigators. Transcatheter aortic-valve replacement with a self-expanding valve in low- risk patients. N Engl J Med 2019;380:1706–15. 30883053 10.1056/NEJMoa1816885

[27] Percy ED , Hirji SA, Kaneko T, Pelletier MP. Patient-reported outcomes: how to advance the minimally invasive debate. J Thorac Cardiovasc Surg 2019;157:e355–6. 30797586 10.1016/j.jtcvs.2019.01.052

[28] Gilmanov D , Bevilacqua S, Murzi M, Cerillo AG, Gasbarri T, Kallushi E et al Minimally invasive and conventional aortic valve replacement: a propensity score analysis. Ann Thorac Surg 2013;96:837–43. 23866805 10.1016/j.athoracsur.2013.04.102

[29] Nair SK , Sudarshan CD, Thorpe BS, Singh J, Pillay T, Catarino P et al Mini-stern trial: a randomized trial comparing mini-sternotomy to full median sternotomy for aortic valve replacement. J Thorac Cardiovasc Surg 2018;156:2124–32.e31. 30075959 10.1016/j.jtcvs.2018.05.057

[30] Glaser N , Persson M, Dalén M, Sartipy U. Long-term outcomes associated with permanent pacemaker implantation after surgical aortic valve replacement. JAMA Netw Open 2021;4:e2116564. 34255050 10.1001/jamanetworkopen.2021.16564 PMC8278270

