# Supplementary material for: Postoperative quality of life and pain after upper hemisternotomy and conventional median sternotomy for aortic valve replacement: results of a randomized clinical trial
Source: Interdiscip Cardiovasc Thorac Surg. 2024 May 15;38(5):ivae083. doi: 10.1093/icvts/ivae083 (PMC11109489; doi:10.1093/icvts/ivae083)
Supplement: ivae083_Supplementary_Data [file ivae083_supplementary_data.doc]

***Supplementary Material***

Supplementary table 1. Baseline characteristics randomized versus prospective registry group.

|  | Randomized group  N=161 | Prospective Registry N=60 | P-value |
| --- | --- | --- | --- |
| Age | 71.6 ± 6.1 | 59.8 ± 10.0 | **<0.001** |
| Female sex | 79 (49.1) | 22 (36.7) | 0.100 |
| Body mass index | 28.1 ± 3.9 | 28.2 ± 5.1 | 0.502 |
| Previous PCI | 7 (4.3) | 2 (3.3) | 0.734 |
| Diabetes | 34 (21.1) | 11 (18.3) | 0.648 |
| - IDDM | 1 (0.6) | 0 (0.0) |  |
| - NIDDM | 33 (20.5) | 11 (18.3) |  |
| Previous stroke | 2 (1.2) | 3 (5.0) | 0.095 |
| Previous TIA | 12 (7.5) | 3 (5.0) | 0.519 |
| COPD | 18 (11.2) | 4 (6.7) | 0.171 |
| - GOLD I | 2 (1.2) | 2 (3.3) |  |
| - GOLD II | 14 (8.7) | 2 (3.3) |  |
| - GOLD III | 2 (1.2) | 0 (0) |  |
| - GOLD IV | 0 (0) | 0 (0) |  |
| Hypertension | 91 (56.5) | 29 (48.3) | 0.277 |
| Pulmonary hypertension | 17 (10.6) | 4 (6.7) | 0.380 |
| - Moderate | 15 (9.3) | 4 (6.7) |  |
| - Severe | 2 (1.2) | 0 (0) |  |
| Kidney function |  |  |  |
| - Normal | 72 (44.7) | 38 (63.3) | **0.014** |
| - Moderate | 80 (49.7) | 22 (36.7) | 0.084 |
| - Severe | 9 (5.6) | 0 (0) | 0.062 |
| NYHA |  |  |  |
| - I | 17 (10.6) | 9 (15.0) | 0.362 |
| - II | 101 (62.7) | 34 (56.7) | 0.411 |
| - III | 40 (24.8) | 17 (28.3) | 0.598 |
| - IV | 3 (1.9) | 0 (0) | 0.287 |
| EuroSCORE I (standard) | 5.87 ± 1.6 | 3.75 ± 1.7 | **0.000** |
| EuroSCORE I (logistic) | 5.27 ± 2.6 | 3.01 ± 1.9 | **0.000** |
| EuroSCORE II | 1.38 ± 0.7 | 0.96 ± 0.5 | **0.000** |

*Values presented as mean ± standard deviation or number of patients (percentage). PCI: percutaneous coronary intervention; IDDM: insulin dependent diabetes mellitus; NIDDM: non-insulin dependent diabetes mellitus; TIA: transient ischemic attack; COPD: chronic obstructive pulmonary disease; NYHA: new york heart association.*

Supplementary table 2. Pre-operative echocardiographic characteristics.

|  | Total group  N=161 | UHS,  N=80 | Conventional SAVR, N=81 | Prospective Registry, N=60 |
| --- | --- | --- | --- | --- |
| LVEF |  |  |  |  |
| - >50% | 138 (85.7) | 72 (90.0) | 66 (81.5) | 53 (88.3) |
| - 31-50% | 21 (13.0) | 8 (10.0) | 13 (16.0) | 4 (6.7) |
| - 21-30% | 2 (1.2) | 0 (0) | 2 (2.5) | 3 (5.0) |
| Aortic valve area, in cm^2^ | 0.80 ± 0.17 | 0.79 ± 0.16 | 0.81 ± 0.18 | 0.85 ± 0.26 |
| Aortic valve mean gradient, in mmHg | 48.1 ± 15.7 | 49.4 ± 16.6 | 46.9 ± 14.8 | 46.5 ± 17.5 |
| Aortic valve jet, in m/s | 4.27 ± 0.62 | 4.29 ± 0.63 | 4.24 ± 0.61 | 4.29 ± 0.63 |
| Aortic regurgitation | 38 (23.6) | 16 (20.0) | 22 (27.2) | 27 (45.0) |
| - Grade I/IV | 18 (11.2) | 9 (11.3) | 9 (11.1) | 14 (23.3) |
| - Grade II/IV | 15 (9.3) | 5 (6.3) | 10 (12.3) | 2 (3.3) |
| - Grade III/IV | 2 (1.2) | 1 (1.3) | 1 (1.2) | 10 (16.7) |
| - Grade IV/IV | 3 (1.9) | 1 (1.3) | 2 (2.5) | 1 (1.7) |

*Values presented as mean ± standard deviation or number of patients (percentage). SAVR: surgical aortic valve replacement; UHS: upper hemisternotomy; LVEF: left ventricular ejection fraction.*

Supplementary table 3. Primary and secondary QoL, per protocol analysis.

|  | **UHS vs Conventional SAVR** | **UHS vs Registry** | **Conventional**  **SAVR vs Registry** | **p-value** |
| --- | --- | --- | --- | --- |
| **Primary outcome (KCCQ)** | **Mean difference across all post-operative time points** | **Mean difference across all post-operative time points** | **Mean difference across all post-operative time points** |  |
| Physical limitations | 2.29 (0.38 to 4.19) | 3.25 (0.70 to 5.79) | 0.95 (-1.49 to 3.40) | **0.016** |
|  |  |  |  |  |
|  | **Mean difference at specific post-operative time points** | **Mean difference at specific post-operative time points** | **Mean difference at specific post-operative time points** |  |
| Month 1 | 3.30 (0.47 to 6.13)* | 5.78 (2.42 to 9.15)* | - |  |
| Month 3 | 2.48 (0.30 to 5.27)* | - | - |  |
| Month 6 | - | - | - |  |
| Month 12 | - | - | - |  |
|  |  |  |  |  |
|  | **Mean difference across all post-operative time points** | **Mean difference across all post-operative time points** | **Mean difference across all post-operative time points** |  |
| Symptoms | 3.31 (0.30 to 6.31) | 3.12 (-0.75 to 6.99) | -0.34 (-4.07 to 3.39) | **0.031** |
|  |  |  |  |  |
|  | **Mean difference at specific post-operative time points** | **Mean difference at specific post-operative time points** | **Mean difference at specific post-operative time points** |  |
| Month 1 | - | - | - |  |
| Month 3 | - | - | - |  |
| Month 6 | 5.46 (1.56 to 9.36)* | - | - |  |
| Month 12 | 4.76 (0.87 to 8.67)* | - | - |  |
|  |  |  |  |  |
| **Secondary outcome (KCCQ)** | **Mean difference across all post-operative time points** | **Mean difference across all post-operative time points** | **Mean difference across all post-operative time points** |  |
| Quality of life | 1.86 (-2.22 to 5.94) | 5.89 (0.55 to 11.25) | 4.04 (-1.09 to 9.17) | 0.096 |
| Social Limitations | 3.90 (0.45 to 7.41) | 6.04 (1.37 to 10.69) | 2.11 (-2.37 to 6.58) | **0.019** |
|  |  |  |  |  |
|  | **Mean difference at specific post-operative time points** | **Mean difference at specific post-operative time points** | **Mean difference at specific post-operative time points** |  |
| Month 1 | 6.70 (1.02 to 12.39)* | 11.24 (4.77 to 17.71)* | - |  |
| Month 3 | - | - | - |  |
| Month 6 | - | - | - |  |
| Month 12 | - | - | - |  |
|  |  |  |  |  |
|  | **Mean difference across all post-operative time points** | **Mean difference across all post-operative time points** | **Mean difference across all post-operative time points** |  |
| Self-Efficacy | 0.03 (-4.09 to 4.15) | 2.57 (-2.85 to 8.01) | 2.55 (-2.66 to 7.76) | 0.58 |
|  |  |  |  |  |
| **Secondary outcome (SF36)** |  |  |  |  |
| Physical Component Summary | 0.87 (-1.19 to 2.95) | 1.38 (-1.32 to 4.09) | 0.51 (-2.08 to 3.11) | 0.55 |
| Mental Component Summary | 0.99 (-0.75 to 2.74) | 2.09 (-0.19 to 4.38) | 1.11 (-1.11 to 3.32) | 0.18 |
|  |  |  |  |  |
| **Postoperative pain** |  |  |  |  |
| Postoperative morphine, in mg | -13.94 (-20.03 to -6.95) | -18.10 (-32.57 to -3.64) | -4.16 (-18.49 to 10.17) | **0.031** |
|  | **Odds ratio** | **Odds ratio** |  |  |
| Pain VAS < 30 mm | 2.79 (1.35 to 5.78) | 3.28 (1.45 to 7.43) | - | **0.006** |

*Mean difference and odds ratio are presented with their respective 95% confidence intervals. The asterisk represents significant differences between groups at a given time point. P-value is the overall p-value between group differences. SAVR: surgical aortic valve replacement; UHS: upper hemisternotomy; KCCQ: Kansas City Cardiomyopathy Questionnaire; SF36: Short Form-36 questionnaire; VAS: visual analogue scale.*

Supplementary table 4. Mean QoL scores for all treatment groups across all time points.

| **UHS versus conventional SAVR versus Registry** | | | |
| --- | --- | --- | --- |
| **Primary Outcome** | **UHS** | **C-SAVR** | **Registry** |
| *Physical Limitation (KCCQ)* |  |  |  |
| - Baseline | 81.2 (1.9) | 82.4 (1.6) | 79.7 (2.7) |
| - Month 1 | 93.6 (1.0) | 90.8 (1.0) | 88.2 (1.2) |
| - Month 3 | 97.5 (1.0) | 95.1 (1.0) | 94.9 (1.3) |
| - Month 6 | 97.1 (1.0) | 95.9 (1.0) | 94.9 (1.3) |
| - Month 12 | 98.8 (1.0) | 96.9 (1.0) | 96.5 (1.3) |
| *Symptoms (KCCQ)* |  |  |  |
| - Baseline | 66.5 (2.3) | 70.6 (2.0) | 65.6 (2.8) |
| - Month 1 | 79.6 (1.4) | 78.8 (1.4) | 78.8 (1.8) |
| - Month 3 | 87.7 (1.4) | 86.3 (1.4) | 84.3 (1.8) |
| - Month 6 | 90.8 (1.4) | 85.8 (1.4) | 85.9 (1.9) |
| - Month 12 | 91.6 (1.4) | 87.0 (1.4) | 89.8 (1.8) |
|  |  |  |  |
| **Secondary Outcome** |  |  |  |
| *Quality of Life (KCCQ)* |  |  |  |
| - Baseline | 65.1 (2.5) | 66.0 (2.1) | 61.2 (3.0) |
| - Month 1 | 73.5 (1.9) | 75.8 (1.9) | 67.4 (2.4) |
| - Month 3 | 88.0 (1.9) | 87.9 (1.9) | 81.3 (2.5) |
| - Month 6 | 91.9 (1.9) | 88.0 (1.9) | 85.0 (2.5) |
| - Month 12 | 94.0 (1.9) | 91.7 (1.9) | 93.6 (2.4) |
| *Social Limitations (KCCQ)* |  |  |  |
| - Baseline | 74.6 (2.6) | 76.8 (2.3) | 71.6 (3.3) |
| - Month 1 | 78.4 (2.1) | 73.7 (2.1) | 69.3 (2.4) |
| - Month 3 | 94.4 (1.8) | 90.2 (1.8) | 89.1 (2.4) |
| - Month 6 | 94.3 (1.8) | 93.4 (1.8) | 90.8 (2.4) |
| - Month 12 | 97.3 (1.8) | 95.9 (1.8) | 95.9 (2.3) |
| *Self-Efficacy (KCCQ)* |  |  |  |
| - Baseline | 51.6 (2.4) | 51.4 (2.3) | 47.6 (3.2) |
| - Month 1 | 50.3 (1.8) | 51.9 (1.8) | 49.2 (2.4) |
| - Month 3 | 50.2 (1.8) | 52.9 (1.8) | 49.3 (2.4) |
| - Month 6 | 51.4 (1.8) | 50.5 (1.8) | 46.8 (2.5) |
| - Month 12 | 50.8 (1.8) | 49.2 (1.8) | 47.5 (2.4) |
| *PCS (SF-36)* |  |  |  |
| - Baseline | 40.8 (1.1) | 41.4 (1.0) | 41.1 (1.3) |
| - Month 1 | 36.6 (0.9) | 37.3 (0.9) | 35.8 (1.2) |
| - Month 3 | 48.0 (0.9) | 47.6 (0.9) | 45.8 (1.2) |
| - Month 6 | 49.1 (0.9) | 48.0 (0.9) | 47.4 (1.2) |
| - Month 12 | 49.6 (0.9) | 48.8 (0.9) | 49.9 (1.2) |
| *MCS (SF-36)* |  |  |  |
| - Baseline | 54.6 (1.1) | 56.2 (1.0) | 50.8 (1.5) |
| - Month 1 | 58.5 (0.8) | 57.9 (0.8) | 55.1 (1.1) |
| - Month 3 | 57.1 (0.8) | 56.7 (0.8) | 55.7 (1.1) |
| - Month 6 | 56.8 (0.8) | 56.3 (0.8) | 55.3 (1.1) |
| - Month 12 | 57.6 (0.8) | 56.4 (0.8) | 57.4 (1.1) |

*Values presented as mean (standard error). C-SAVR: conventional surgical aortic valve replacement; UHS: upper hemisternotomy; KCCQ: Kansas City Cardiomyopathy Questionnaire; SF-36: Short Form 36; PCS: Physical Component Summary; MCS: Mental Component Summary.*

Supplementary table 5. Mean postoperative pain scores for all treatment groups, per protocol analysis.

| **UHS versus conventional SAVR versus Registry** | | | |
| --- | --- | --- | --- |
| Postoperative VAS | C-SAVR | UHS | Registry |
| Day 1 | 39.7 (2.2) | 28.6 (2.3) | 35.4 (2.6) |
| Day 2 | 32.4 (2.2) | 24.7 (2.3) | 36.8 (2.5) |
| Day 3 | 23.6 (2.2) | 18.2 (2.3) | 25.3 (2.6) |
| Day 4 | 21.7 (2.2) | 16.3 (2.3) | 23.8 (2.7) |
| Day 5 | 17.7 (2.2) | 13.3 (2.3) | 17.8 (2.8) |
| Day 6 | 15.1 (2.3) | 12.8 (2.4) | 11.2 (2.9) |
| Day 7 | 14.3 (2.6) | 15.3 (2.7) | 8.2 (4.1) |

*Values presented as mean (standard error). C-SAVR: conventional surgical aortic valve replacement; UHS: upper hemisternotomy; VAS: visual analogue scale.*

|  | Total group  N=157 | UHS,  N=73 | Conventional SAVR, N=84 | Prospective  Registry, N=60 | P-value  UHS vs Conventional SAVR |
| --- | --- | --- | --- | --- | --- |
| CPB time, in min | 72.1 ± 25.4 | 71.7 ± 19.5 | 72.3 ± 29.7 | 95.7 ± 31.5 | 0.897 |
| ACC time, in min | 48.6 ± 19.8 | 48.8 ± 15.6 | 48.4 ± 22.9 | 68.2 ± 22.6 | 0.891 |
| Total time surgery, in min | 148.9 ± 53.9 | 154.6 ± 32.6 | 144.9 ± 66.8 | 168.7 ± 42.4 | 0.262 |
| Total time OR, in min | 210.2 ± 55.6 | 216.5 ± 34.5 | 205.4 ± 68.6 | 223.6 ± 46.3 | 0.211 |
| Blood loss, in ml | 443 ± 319 | 347 ± 132 | 528 ± 402 | 445 ± 235 | **0.000** |
| Postoperative transfusion | 7 (4.5) | 4 (5.5) | 3 (3.6) | 1 (1.7) | 0.689 |
| Ventilation on ICU, hours | 5.6 ± 3.8 | 4.8 ± 3.9 | 6.2 ± 3.7 | 5.11 ± 3.2 | 0.103 |
| Length of stay, days | 8 [6 – 10] | 8 [6 – 11] | 7 [6 – 10] | 7 [6 – 8] | 0.985 |
| 30-day mortality | 3 (1.9) | 0 (0) | 3 (3.6) | 1 (1.7) | 0.103 |
| Stroke | 3 (1.9) | 2 (2.5) | 1 (1.2) | 1 (1.7) | 0.598 |
| Myocardial infarction | 0 (0) | 0 (0) | 0 (0) | 0 (0) | - |
| Acute kidney injury | 9 (5.7) | 5 (6.8) | 4 (4.8) | 0 (0) | 0.575 |
| LCOS | 3 (1.9) | 1 (1.4) | 2 (2.4) | 2 (3.3) | 1.000 |
| Endocarditis | 1 (0.6) | 0 (0) | 1 (1.2) | 0 (0) | 1.000 |
| Permanent pacemaker | 14 (8.9) | 8 (11.0) | 6 (7.1) | 3 (5.0) | 0.403 |
| Third degree AVB | 19 (12.1) | 11 (15.1) | 8 (9.5) | 4 (6.7) | 0.288 |
| Sepsis | 4 (2.5) | 0 (0) | 4 (4.8) | 0 (0) | 0.124 |
| Multi-organ failure | 2 (1.3) | 0 (0) | 2 (2.4) | 0 (0) | 0.499 |
| Major vascular complication | 1 (0.7) | 0 (0) | 1 (1.2) | 0 (0) | 1.000 |
| Sternal dehiscence | 2 (1.3) | 1 (1.3) | 1 (1.2) | 0 (0) | 1.000 |
| Mediastinitis | 3 (1.9) | 1 (1.3) | 2 (2.4) | 0 (0) | 1.000 |
| Severe paravalvular leak | 2 (1.3) | 1 (1.3) | 1 (1.2) | 0 (0) | 1.000 |
| Readmission ICU | 2 (1.3) | 1 (1.3) | 1 (1.2) | 0 (0) | 1.000 |
| Tamponade | 7 (4.5) | 5 (6.8) | 2 (2.4) | 9 (15.0) | 0.176 |
| Reoperation for bleeding | 3 (1.9) | 0 (0) | 3 (3.6) | 0 (0) | 0.249 |
| New onset AF | 51 (32.5) | 28 (38.4) | 23 (27.4) | 12 (20.0) | 0.173 |
| Left bundle branch block | 50 (31.8) | 24 (32.9) | 26 (31.0) | 6 (10.0) | 0.796 |
| Right bundle branch block | 4 (2.5) | 3 (3.8) | 1 (1.2) | 2 (3.3) | 0.338 |

Supplementary table 6. Secondary surgical outcome, per protocol analysis.

*Values presented as mean ± standard deviation, median and interquartile range or number of patients (percentage). P-value is the result of UHS compared with conventional SAVR. SAVR: surgical aortic valve replacement; UHS: upper hemisternotomy; CPB: cardiopulmonary bypass; ACC: aortic cross clamping; OR: operating room; ICU: intensive care unit; LCOS: low cardiac output syndrome; AVB: atrioventricular block; AF: atrial fibrillation.*
